# Tetrahydrocannabivarin is Not Tetrahydrocannabinol

**DOI:** 10.1089/can.2024.0051

**Published:** 2025-02-13

**Authors:** Mehdi Haghdoost, Erica N. Peters, Matthew Roberts, Marcel O. Bonn-Miller

**Affiliations:** ^1^ Nalu Bio Inc, San Francisco, California, USA.; ^2^ Emerald Mountain Consulting, LLC, Charlottesville, Virginia, USA.; ^3^ Charlotte’s Web, Louisville, Colorado, USA.

**Keywords:** THCV, THC, pharmacology, mechanisms of action

## Abstract

Tetrahydrocannabivarin (THCV) is a phytocannabinoid that is becoming popular across the North American cannabis market. THCV has been reported to reduce blood sugar and act as an appetite suppressant in several independent pre-clinical studies, which has earned it the popular nickname of “diet weed,” despite few human studies of these effects. Additionally, THCV is usually and incorrectly categorized as an intoxicating analogue of tetrahydrocannabinol (THC), which causes confusion among both consumers and regulators. In this article, we examine what is known pre-clinically and clinically about THCV, as well as highlight mechanisms of action, in order to clarify the scientific differences between THCV and THC. THCV, although structurally similar to THC, has distinct pharmacological activity and physiological effects at the doses currently reported in the literature. We highlight areas of opportunity for further THCV research in order to determine the full and appropriate potential for unique health, wellness, and therapeutic applications of this compound.

## Introduction

**FIG. 1.**
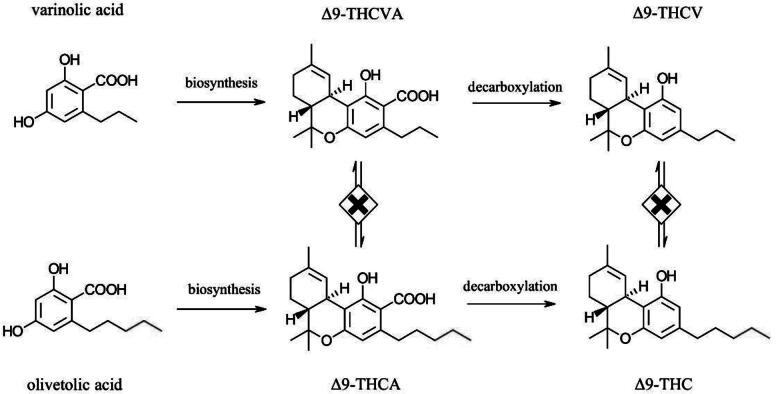
Biosynthesis of △9-THCV and △9-THC from varinolic and olivetolic acids, respectively. THCV, tetrahydrocannabivarin; THC, tetrahydrocannabinols.

Tetrahydrocannabivarins (THCVs) are not tetrahydrocannabinols (THCs) nor are they THC isomers. There is no known biosynthesis pathway in the cannabis plant nor any synthetic chemical reaction that can convert THC to THCV or *vice versa*. In the cannabis plant, tetrahydrocannabivaric acid (THCVA) originates from varinolic acid. In contrast, olivetolic acid is the origin of tetrahydrocannabinolic acid (THCA) (Fig. 1). After biosynthesis, natural decarboxylation, which can be accelerated with heat,^1^ converts the acidic precursors THCVA and THCA to neutral THCV and THC, respectively. The length of the alkyl chain is the main structural difference between these two precursors, which leads to THCV having a smaller molecular weight than THC. The shorter alkyl tail of THCV is meaningful enough to give it distinct chemical, physical, pharmacological, and physiological properties relative to THC and other phytocannabinoids. Despite these scientifically well-documented differences, THC and THCV are commonly confused with one another. It is, therefore, important to review the relevant scientific reports to provide a clear understanding of what we know to be the difference between these two important phytocannabinoids and to point to the distinct therapeutic potential of each.

**FIG. 2.**
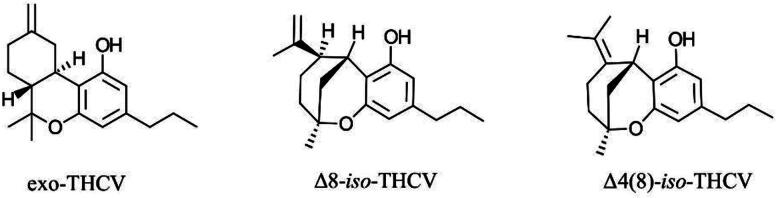
Other THCV isomers that can rise from synthetic or semi-synthetic approaches to Δ8-THCV. THCV, tetrahydrocannabivarin.

One factor possibly contributing to the confusion between THCV and THC is the presence of two common isomers for THCV: Δ9-THCV and Δ8-THCV, similar to the two common isomers for THC, Δ9-THC and Δ8-THC. The Δ9 isomer of THCV, a phytocannabinoid first discovered in *Cannabis sativa* by Merkus in 1971^2^ is commonly extracted from THCV-dominant cultivars. Conversely, no report mentions the presence of Δ8-THCV in cannabis plants, suggesting that this isomer is a semi-synthetic cannabinoid made by simple acid isomerization of cannabidivarin (CBDV). However, as THCV-dominant *C. sativa* cultivars are rare, it could also be that no investigation so far has targeted the detection of Δ8-THCV in the plant material. *In vitro* results suggest that Δ9-THCV and Δ8-THCV are both cannabinoid CB1 receptor (CB1R) antagonists, with Δ8-THCV having around ∼2X less potency than Δ9-THCV.^3^ To our knowledge, human studies to support this difference in potency between THCV isomers are absent. It is noteworthy that the semi-synthetic pathway to Δ8-THCV can result in other THCV isomers such as Δ9 (11)-THCV (exo-THCV), Δ8-*iso*-THCV, and Δ4 (8)-*iso*-THCV (Fig. 2).^4–6^ The biological activity of these isomers has not been investigated, and owing to the lack of robust analytical methods for their quantitative analysis, their presence in THCV products has not been studied and they are most often generally identified simply as Δ8-THCV. The overlap in terminology and the lack of detailed analysis of various THCV isomers, as described, can lead to confusion regarding the precise composition and potential effects of THCV products, making it challenging for consumers and researchers to understand what specific compounds they are dealing with.

Despite the similarity in the isomers for both THCV and THC, *in vitro* receptor binding research supports that THCV can be distinguished from THC because of differential CB1R binding behavior. As reviewed by Pertwee,^7^ THC is a known potent partial agonist of CB1R, with *K*i = 5–80 nM. Functionality of ligands at CB1R, particularly agonism, is required for creating the cannabis intoxication effect associated with THC.^8^ THCV has a similar ring structure to THC, but it has a chain length of 3 carbons instead of 5. Although THCV can bind to CB1R with high affinity (Ki = 22–75.4 nM),^9,10^ THCV is not a CB1R agonist but instead a CB1R antagonist at most biologically relevant dosages. THCV antagonism of CB1R has been highlighted in multiple studies. In one of the most recent studies, Walsh and Holmes report that Δ9- and Δ8-THCV antagonized the CB1R in an isomer- and ligand-dependent manner.^3^

The reason behind the difference in biological properties between THC and THCV is poorly understood. An article by Raïch and coworkers suggests that the difference in the functionality of Δ9-THC and Δ9-THCV at CB1R originates from variation in binding modes that results in qualitatively different effects depending on the signaling pathway engaged upon receptor activation.^11,32^ However, the functionality of THCV at CB1R appears to be complex, as some studies have highlighted weak CB1R agonist properties for Δ9-THCV. Pertwee suggests that Δ9-THCV behaves mainly as a CB1R antagonist but, at higher doses, Δ9-THCV acts as a CB1R agonist.^7^ Indeed, one study has reported Δ9-THCV as a weak partial agonist in the CB1R cAMP inhibition assay.^7^ Even weak CB1R agonist activity at high concentrations of Δ9-THCV may lead one to expect intoxicating effects in humans at elevated doses; however, accurately predicting such effects from *in vitro* data is challenging owing to the concentration-dependent dual agonist/antagonist behavior of THCV.

A few *in silico* studies have also investigated differences in CB1R binding behavior between THC and THCV. In a pre-print work at arXiv, Shahbazi and colleagues use molecular docking calculation to show that Δ9-THC binding to CB1R leads to a larger shift in the helices position of the receptor relative to Δ9-THCV binding (switch toggle theory).^12^ Molecular dynamics and docking studies by Torrent et al. suggest that conformation adopted by the alkyl chain of Δ9-THC and Δ9-THCV inside the receptor orthosteric site is likely the reason for their difference in CB1R functionality.^13^ Further studies are required to fully understand the complexity and to validate this theory. It is also still not understood why THCV has shown dose-dependent dual agonist/antagonist behavior in some studies.^14,15^

We found very limited scientific data in our attempt to compare the activity of THCV and THC at other human receptors. The agonist property of THC (both Δ8 and Δ9) at the CB2 receptor (CB2R) is very well established.^16^ THCV is reported to have a weaker affinity toward CB2R than THC (reviewed to some extent here^7^). However, similar to CB1R, some studies have reported THCV as a potent agonist^17^ of CB2R and some as an antagonist.^9^ In addition, although both Δ9-THC and Δ9-THCV are reported to affect the serotonergic pathway, particularly through the 5-HT_1A_ receptor,^18,19^ the absence of side-by-side comparison prevents us from drawing a solid conclusion about functional similarities and differences. Finally, THC and THCV are similarly active at transient receptor potential (TRP) channels such as TRPV1, TRPV2, TRPV3, TRPV4, TRPA1, and TRPM8 (for a review on this topic, see Muller et al.^20^) with THC being relatively more potent than THCV. However, this similarity is hardly of any significance as almost all phytocannabinoids, intoxicating or not, have some activity at TRP receptors.

Given their differential behavior at CB1R, one area of study has focused on the interaction between THC and THCV. As a competitive antagonist to CB1R agonists, THCV is predicted to act as a competitive antagonist of THC and could inhibit the effects of THC in a dose-dependent fashion in humans and animals. Preliminary scientific data appear to support this hypothesis, at least at specific doses of THCV. Pertwee and coworkers reported that at intravenous doses of 0.1–3 mg, Δ9- and Δ8-THCV attenuated Δ9-THC-induced anti-nociception (tail-flick test) and hypothermia (rectal temperature) in rats, and Δ8-THCV also antagonized Δ9-THC-induced ring immobility.^10^ In a recent drug discrimination study,^21^ Δ8-THCV (10–100 mg/kg, i.p.) did not alter the discriminative stimulus effects of Δ9-THC in male and female rats trained to discriminate Δ9-THC from vehicle. However, Δ8-THCV showed a weak signal in modulating the Δ9-THC effect by decreasing response rates (response/sec).^21^ In humans, 10 mg of oral pure Δ9-THCV administered daily for five days, relative to placebo, inhibited some of the effects of 1 mg intravenous Δ9-THC, such as delayed verbal recall, heart rate, and subjective intensity of effects, although it also increased memory intrusions.^22^ The findings of this study may have limited ecological validity, however, as Δ9-THC was administered intravenously rather than *via* inhalation or ingestion. The antagonist effect of THCV isomers does not seem to be limited to THC; pre-clinical studies show that THCV isomers are also capable of antagonizing potent full agonists of CB1R, such as WIN55212.^3^ The antagonism effect of Δ9-THCV is, however, ∼3 and ∼45 times less than AM-281 and rimonabant, respectively (based on IC_50_ values).

A few human studies of THCV have provided insights into differences in subjective effects relative to THC. There are two human THCV studies that have demonstrated that 10 mg oral Δ9-THCV may be therapeutic in the treatment of obesity *via* impacts on blood sugar and appetite^23,24^ (for a review on potential therapeutic benefits of THCV in the management of obesity and diabetes, see Abioye and colleagues^25^). The appetite-suppressing effect of Δ9-THCV is in stark contrast to Δ9-THC, which is known to increase appetite and craving for food.^26^ Additional literature on the effects of THCV in humans is scarce. Early human studies show that the effects of THC and those of THCV at similar doses are quite different. For example, a 10 mg oral dose of Δ9-THC reliably produces an intoxicating effect in humans,^27^ which has been used to inform product limits in individual U.S. state markets (e.g., Colorado^28^) and in other countries (e.g., Canada^29^). However, the subjective effects of 10 mg of oral Δ9-THCV, when administered as a single dose (5 mg twice daily)^23^ or as a repeated dose over 5 days,^22^ have been indistinguishable from those of placebo. It is worth noting that most THCV consumer products contain less than 5 mg THCV. Indeed, much higher oral doses of THCV are needed to produce subjective effects in humans. In a recent placebo-controlled, within-subjects human study on acute doses of Δ8-THCV ranging from 12.5 to 200 mg, 12.5 mg, 25 mg, and 50 mg doses of Δ8-THCV did not demonstrate subjective or cognitive effects typically associated with Δ9-THC consumption.^30^ Very slight Δ9-THC-like effects were observed at 100 and 200 mg doses of Δ8-THCV, based in part on a mean observed rating of 0.67 (100 mg Δ8-THCV) and 1.04 (200 mg Δ8-THCV) on the “Marijuana” scale of the Addiction Research Center Inventory (ARCI).^30^ As a point of comparison, 3× to 5× higher ARCI Marijuana scores have been observed after acute dosing of less than 10 mg of Δ9-THC.^31^ It is unclear how the effects observed for Δ8-THCV translate to the Δ9-THCV isomer, but findings appear to suggest that the subjective Δ9-THC-like effects produced by high oral doses of Δ8-THCV are of low magnitude.

In conclusion, although close in chemical structure, THCV and THC show distinct pharmacological and physiological properties and are produced through distinct biosynthetic pathways. Several published scientific studies have concluded THCV to be an antagonist of CB1R, whereas it is widely accepted that THC is a potent partial agonist for this same receptor. Early human studies indicate that low oral doses (10 mg) of Δ9-THC elicit an intoxicating effect, whereas similar doses of Δ9-THCV do not. Only at much higher oral doses of Δ8-THCV (100 and 200 mg) have any cannabis-like effect been documented, though at a considerably lower magnitude than has been demonstrated with low doses (<10 mg) of Δ9-THC. Although available evidence points to THCV mainly inhibiting the effects of THC, human pharmacokinetic and pharmacodynamic studies of other doses of THCV with oral doses of THC are needed. Based on this perspective of scientific data, we believe that THCV and THC should not be considered in the same category of psychoactive compounds and should not be grouped together from a regulatory standpoint. We also note the potential difference in antagonist potency of Δ9-THCV and Δ8-THCV, suggesting that one of the next steps in THCV research can be devoted to understanding the difference in dose-effect of these isomers in humans.

## Notes

### Abbreviations Used

Δ4(8)-iso-THCV: Delta-4(8)-iso-tetrahydrocannabivarin
Δ8-iso-THCV: Delta-8-iso-tetrahydrocannabivarin
Δ8-THC: Delta-8-tetrahydrocannabinol
Δ8-THCV: Delta-8-tetrahydrocannabivarin
Δ9-THC: Delta-9-tetrahydrocannabinol
Δ9-THCV: Delta-9-tetrahydrocannabivarin
Δ9(11)-THCV: Delta-9(11)-Tetrahydrocannabivarin
ARCI: Addiction Research Center Inventory
CB1R: Cannabinoid CB1 receptor
CB2R: Cannabinoid CB2 receptor
CBDV: Cannabidivarin
IC50: Half maximal inhibitory concentration
i.p.: Intraperitoneal
i.v.: Intravenous
Ki: Inhibition constant
THC: Tetrahydrocannabinol
THCA: Tetrahydrocannabinolic acid
THCV: Tetrahydrocannabivarin
THCVA: Tetrahydrocannabivaric acid
TRP: Transient receptor potential
TRPA1: Transient receptor potential ankyrin 1
TRPM8: Transient receptor potential melastatin 8
TRPV1: Transient receptor potential vanilloid 1
TRPV2: Transient receptor potential vanilloid 2
TRPV3: Transient receptor potential vanilloid 3
TRPV4: Transient receptor potential vanilloid 4
